# Porcine vs bovine surfactant therapy for preterm neonates with RDS: systematic review with biological plausibility and pragmatic meta-analysis of respiratory outcomes

**DOI:** 10.1186/s12931-019-0979-0

**Published:** 2019-02-06

**Authors:** Ascanio Tridente, Lucia De Martino, Daniele De Luca

**Affiliations:** 1 Department of Molecular and Clinical Pharmacology, University of Liverpool, Liverpool, UK; 2 Critical Care Unit, Whiston Hospital, St Helens and Knowsley Teaching Hospitals, Merseyside, UK; 3 Division of Pediatrics and Neonatal Critical Care, Medical Center “A. Béclère”, South Paris University Hospitals, Assistance Publique–Hôpitaux de Paris (APHP) , Paris, France; 4 Physiopathology and Therapeutic Innovation Unit, INSERM U999, South Paris-Saclay University, Paris, France

**Keywords:** RDS, Neonate, Surfactant, Therapy

## Abstract

**Background:**

Bovine surfactants are known to be clinically equivalent but it is unclear if porcine or bovine surfactants *at their licensed dose* should be preferred to treat respiratory distress syndrome in preterm neonates.

**Methods:**

We performed a comprehensive review of biochemical and pharmacological features of surfactants to understand the biological plausibility of any clinical effect. We then performed a pragmatic meta-analysis comparing internationally marketed porcine and bovine surfactants for mortality and respiratory outcomes. Search for randomised controlled trials with no language/year restrictions and excluding “grey” literature, unpublished or non-peer reviewed reports was conducted, following Preferred Reporting Items for Systematic Reviews and Meta-Analyses guidelines and the most recent methodological recommendations.

**Results:**

Sixteen articles were included in the review and 14 in the meta-analysis (1491 neonates). 200 mg/kg poractant-α (a porcine surfactant) was associated with lower BPD/mortality (OR 0.632[95%CI:0.494, 0.809]*;p* < 0.001),BPD (OR 0.688[95%CI:0.512, 0.925];*p* = 0.013), retreatment (OR 0.313[95%CI:0.187, 0.522];*p* < 0.0001), airleaks (OR 0.505[95%CI:0.308, 0.827];*p* = 0.006) and lung haemorrhage (OR 0.624[95%CI:0.388, 1];*p* = 0.051). Gestational age is associated with effect size for BPD (coefficient: 0.308 [95%CI:0.063, 0.554];*p* = 0.014) and surfactant retreatment (coefficient: -0.311 [95%CI:-0.595, − 0.028];*p* = 0.031).

**Conclusion:**

200 mg/kg poractant-α is associated with better respiratory outcomes compared to bovine surfactants *at their licensed dose*. The effect of poractant-α on BPD and surfactant retreatment is greater at lowest and highest gestational ages, respectively.

**Trial registration:**

PROSPERO n.42017075251.

**Electronic supplementary material:**

The online version of this article (10.1186/s12931-019-0979-0) contains supplementary material, which is available to authorized users.

## Background

Respiratory distress syndrome (RDS) is the main cause of respiratory failure in preterm neonates and its incidence differs depending on gestational age and birth weight [1]. RDS was originally known as “hyaline membrane disease”, based on its histological appearance [2] and re-named RDS after the acceptance that it was caused by primary surfactant deficiency [3].

When optimal prenatal care is provided, the best approach to treat RDS, according to several recent trials, [4] consists in providing continuous positive airway pressure from the first minutes of life using short binasal prongs, [5] followed by early selective surfactant administration for babies with worsening oxygenation and/or increasing work of breathing. Both European and American guidelines advise in favour of this strategy, which reduces mortality and broncho-pulmonary dysplasia (BPD) [6, 7]. Nevertheless, it remains unclear whether the use of different surfactants might influence the outcomes. Currently available surfactants are animal-derived preparations. They resulted to be superior to older synthetic (protein-free) surfactants, as the proteins improve surfactant activity, stabilizing the film at the air/liquid interface [8]. Animal-derived surfactants may carry pharmacological and biochemical differences and these latter might influence clinical outcomes.

A 2015 Cochrane meta-analysis subdivided surfactants in bovine and porcine-derivedand according to the extraction method (lung lavage or minced lung extract): [9] neonates treated with porcine minced surfactant had more favourable outcomes than those treated with bovine minced lung surfactant. The meta-analysis did not identify any significant difference between bovine lung lavage and bovine minced lung-derived surfactant [9]. Other comparisons between different surfactants were either based on a single trial or not feasible, due to lack of studies [9].

Our aim was to: 1) comprehensively review all the pharmacological and biochemical differences between available animal-derived surfactants, and 2) compare porcine and bovine surfactants with regard to mortality and respiratory outcomes in preterm neonates with RDS.

Such a pragmatic meta-analysis was limited to randomised controlled trials investigating the use of internationally available surfactants; since no significant differences have been detected between bovine surfactants of different extraction method (minced or lung lavage), [9] porcine surfactants were compared to all bovine surfactants, irrespective of their method of preparation.

## Methods

### Protocol

Prior to commencing the search, a systematic review protocol was agreed to determine the databases to be searched, search modality, eligibility criteria, data extraction/aggregation methodology, timing of meetings and methods for dispute resolution in case of disagreement. Following the agreement, this review was registered in the international prospective register of systematic review (PROSPERO n.42017075251). Regular meetings between the authors were scheduled and the Preferred Reporting Items for Systematic Reviews and Meta-Analyses guidelines were followed through the entire project [10].

### Review of the pharmacological and biochemical surfactant features

We obtained pharmacological and biochemical data from previous publications in the field [11–15] and by systematically looking within the product leaflets and the manufacturer websites. When in doubt or when informations were lacking, we contacted directly the manufacturer or, in case of no answer, the dealer and/or authors of related papers. At least two e-mails have been sent before writing to the alternative contact.

### Eligibility criteria

We looked for randomized controlled trials fulfilling the following criteria: 1) published as full articles or as abstracts presented at the Paediatric Academic Societies (PAS) or European Society for Paediatric Research (ESPR) meetings; 2) enrolled preterm neonates (gestational age < 37 weeks) with clinical and/or radiological established evidence of RDS needing intubation; 3) compared porcine and bovine-derived surfactants (irrespective of their preparation method); and 4) reported at least one of the selected outcomes (see below).

Studies fulfilling these criteria were finally included in the meta-analysis, if they compared surfactants internationally available on the market. Since early selective surfactant treatment is currently advised by international guidelines, [6, 7] we did not consider trials on surfactant prophylaxis (i.e. with surfactant administered without any evidence of RDS, in the first minutes of life). No language or year restrictions were applied. We excluded “grey” literature, unpublished or non-peer reviewed reports.

### Information sources and search strategy

We conducted a literature search (on June 25, 2018) of the following databases: AMED, BNI, CINAHL, EMBASE, HBE, HMIC, Medline, PsycINFO, PubMed, using the NICE National Institute for Healthcare Excellence Healthcare Databases Advanced Search portal. We used the following as key words and/or MeSH terms: “treatment”, “bovine lipid extract surfactant”, “BLES”, “beractant”, “survanta”, “surfacen”, “surfactant-TA”, “surfacten”, “bovactant”, “alveofact”, “calfactant”, “infasurf”, “poractant alfa”, “curosurf”, “newfactant”. We searched the abstract archives of the PAS and ESPR meetings and the clinicaltrials.gov registry. We also hand-searched references cited in the studies identified through the initial search, review articles on the subject and the authors’ personal archives. Finally, we contacted experts in the field and letters commenting the trials have also been reviewed.

We used the following string: (treatment AND ((((bovine AND lipid) AND extract) AND surfactant) OR BLES OR beractant OR survanta OR surfacen OR surfactant-TA OR surfacten OR bovactant OR alveofact OR calfactant OR infasurf OR poractant alfa OR curosurf OR newfactant)).ti,ab.

### Study selection

Details of all studies retrieved were included in a database, removing duplicates. All authors reviewed abstracts, and (where necessary) full text of the remaining articles, excluding those not meeting the eligibility criteria.

### Data collection process

We developed a data extraction sheet (based on the Cochrane Consumers and Communication Review Group’s data extraction template), pilot-tested it on three randomly-selected studies, and refined it accordingly. Data from included trials were extracted independently by two authors (AT, DDL) and then cross-verified. Discrepancies were resolved through discussion between the two reviewers and, if no agreement could be reached, with the third investigator. Where further clarification was needed or when data could not be statistically aggregated authors were contacted to provide clarification and/or raw data.

### Data items

Data collected included study design, number of enrolled patients, prenatal corticosteroid, mean gestational age, inclusion and exclusion criteria, surfactant type and doses, outcomes and variables used to assess study quality.

The outcomes were: 1) in-hospital mortality; 2) BPD defined as need for supplemental oxygen at postmenstrual age of 36 weeks or, if this latter was unavailable, at 28 days of postnatal age; 3) composite BPD/mortality endpoint; 4) air leaks defined as occurrence of pneumothorax, pneumomediastinum and/or pulmonary interstitial emphysema occurring after surfactant administration; 5) surfactant re-treatment; 6) lung haemorrhage defined as bright red blood in the endotracheal tube, with rapid deterioration of the clinical and/or respiratory status, occurring after surfactant administration.

### Assessment of risk of Bias

The Cochrane Risk of Bias assessment tool [16] was used to evaluate the study quality. Two reviewers (AT,DDL) independently assessed the risk of bias for each trial, including: 1) selection bias (inadequate random sequence generation, failure to conceal treatment allocation); 2) performance bias (inadequate blinding of patients and investigators/personnel); 3) detection bias (failure to adequately blind the outcome assessors); 4) attrition bias (incomplete outcome data evaluation and failure to follow intention-to-treat analysis); 5) reporting bias (selective outcome reporting); 6) any other bias and any potential conflict of interest.

Each item was assessed as at “low” or “high risk” of bias, or unclear (when the authors were unable to determine, on the basis of the available information). Discrepancies were resolved through discussion between the two reviewers and, if no agreement could be reached, with the third investigator. The presence of publication bias was explored through: 1) visual assessment of a Funnel plot; 2) Egger regression and 3) Peters’ test, according to recent published recommendations [17]. More details are available in the Additional file 1.

### Summary measures and synthesis of results

We performed the following analyses: 1) 200 mg/kg poractant-α versus 100 mg/kg bovine surfactants; and 2) any dose of poractant-α versus 100 mg/kg bovine surfactants (that is, pooling the data from all treatment arms in which poractant-α was administered, irrespective of the dosage used). We did not perform a separate analysis of 100 mg/kg poractant-α versus 100 mg/kg bovine surfactants, since this would have been unreliable because only based on 2–3 trials and smaller patient populations.

Outcomes were analysed using weighted average odds ratios (OR) and 95% confidence interval (95% CI) for all outcomes. We used the DerSimonian-Laird random-effects model and inverse variance method. Such approach is more conservative than the fixed effect model as, it assumes the presence of heterogeneity among aggregated studies, based on the assumption that the studies considered are estimating different underlying effect sizes [18]. Consistency across the studies was evaluated using the *I*^2^ statistic (variation in ORs attributable to heterogeneity) and performing a χ^2^ test for heterogeneity; an *I*^2^ value greater than 50% was considered as indicative of substantial heterogeneity.

### Additional analyses

Antenatal steroid prophylaxis might be a relevant confounding bias for our outcomes, as it boosts the production of endogenous surfactant [19]. Studies have been published across several years (from 1995 to 2017), that is also before the widespread use of prenatal steroids in common clinical practice: thus, steroids have been variously administered amongst the studies. RDS is also known to be more severe at lower gestational ages [20, 21] and this may be another confounder.

Finally, poractant-α dose can be a confounder, since this is the only surfactant with two licensed doses and can be administered at 100 or 200 mg/kg. Thus, we performed three random-effects model meta-regressions per each clinical outcome [22] and we inserted as covariates: 1) the use of antenatal steroids (as % of neonates treated in each study), 2) the gestational age (as mean gestational age (in weeks) of each study population), and 3) the dose of poractant-α administered in each study. We only used one covariate in each model in order to reduce false positive conclusions [22]. Coefficients (and 95%CI) have been reported for each covariate. All analyses were performed with SPSS 15.0 (SPSS inc, Chicago, IL, USA), Open-MetaAnalyst 10.1 [23] and Meta-essentials [24].

## Results

Table 1 describes the biochemical and pharmaceutical features of all animal-derived surfactants currently available to treat RDS in preterm neonates. There is a wide variation in phospholipid profile amongst surfactants. Surfactant-protein content is also variable and poractant-α is the preparation with the highest protein concentrations (especially of surfactant protein-B). All surfactant but poractant-α have similar formulation in terms of phospholipid concentration (roughly about 30 mg/mL). Poractant-α shows a concentration of 80 mg/mL.

**Table 1:** Biochemical and pharmacological data of all current animal-derived surfactant preparations

	Biochemical (trade) name	Preparation method	Total PL (mg/mL)	Main PL°	SP-B ∫ (mg/mL)	SP-C ∫ (mg/mL)	Dose # (mg/mL)	Volume (mL/kg)
Bovine	Beractant (Survanta®)	Enriched minced lung†	25	DPPC (70%) and PS (4%)	0.03	0.3	100	4
	BLES (Neosurf® or Liposurf®)	Lung lavage	27	DPPC (42%) and PG (11%)	0.17	0.49	135	5
	Bovactant (Alveofact®)	Lung lavage	45	DPPC (39%) and PG (8.5%)	0.3	0.7	50	1.2
	Calfactant (Infasurf ®)	Lung lavage (from calves)	35	DPPC (41%) and PG (6%)	0.26	0.36	105	3
	Calf Pulmonary Surfactant for Injection (Kelisu®) ^$^	Lung lavage (from calves)	30	DPPC 48% other data n.a.	0.2	0.25	100	3.3
	Korean bovine surfactant (Newfactan ®)	Minced lung	25	DPPC (60%) and PG (6%)	@	@	120	4.8
	Surfactant-TA (Surfacten®)	Enriched minced lung†	25	DPPC (65%) other data n.a.	n.a.	n.a.	120	4.8
Porcine	Butantan^$^	Minced lung	25	DPPC (76%) and PE (7%)	^	^	100	4
	Poractant-α (Curosurf ®)	Modified minced lung*	80	DPPC (46%) and PE (6%)	0.45	0.9	100 or 200	1.25 or 2.5
	Surfacen^$^	Lung lavage	25	DPPC (45%) and PI (12%)	π	π	100	4

Data have been rounded to the closest decimal; some data represent an average of the different values available and should be considered as an estimation. **$**Kelisu, Butantan and Surfacen are only marketed in China, Brasil and Cuba, respectively. **†**Minced lung is subjected to DPPC addition and the preparation method is analogous, which makes these two surfactants similar. **@**All surfactant proteins in Newfactant represent 1.1 ± 0.17 of the total. **^**Protein B and C in Butantan represent 5–10% of the total; **π**All surfactant proteins in Surfacen represent 1% of the total. No more details were available for these three surfactants (Newfactant, Butantan and Surfacen); protein content in Newfactant, Butantan and Surfacen is not expressed in mg/mL of the final solution, thus it is not comparable to that of other surfactants. The manufacturer of surfactant-TA refused to disclose additional details. *Modification consists in concentration by liquid gel chromatography. **°**DPPC is expressed as % of PC (with the exception of Kelisu where it is expressed as % of the total phospholipids), while other phospholipids are expressed as % to the total mass of surfactant. **∫** Surfactant proteins are expressed as mg/mL of final surfactant solution in the vial, normalized for the DPPC concentration (with the exception of Newfactant, Butantan and Surfacen – see above). **#**This is intended as the licensed dose for surfactants commercially available on the international market and as a suggested dose for Butantan and Surfacen which are available in Brazil and Cuba, respectively. *Abbreviations*: *PL* phospholipids, *SP-B* surfactant protein-B, *SP-C* surfactant protein-C

**Fig. 1:**
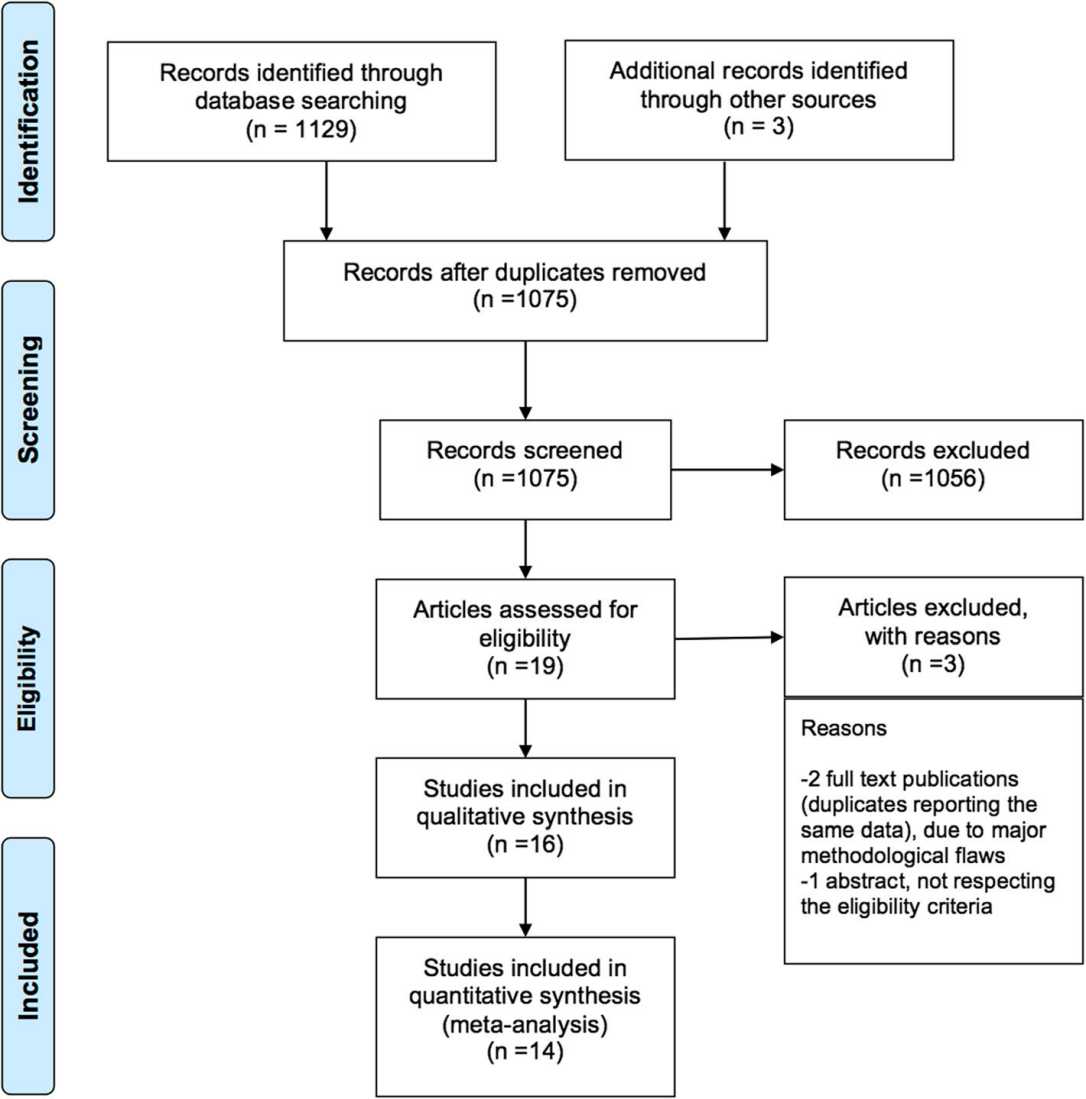
Flow chart of the review. The studies excluded from the systematic review were two full text duplicates [24, 25] reporting the same data with major methodological flaws (lack of randomization, unclear analysis, lack of allocation concealment and blinding, unclear sample size calculation, unclear outcome definition and incomplete outcome analysis/reporting) and one abstract which did not respect the eligibility criteria [22]. Two studies [42, 43] included in the systematic review were excluded from the meta-analysis because they investigated the use of non-internationally available porcine surfactants

Figure 1 illustrates the study selection for the meta-analysis: three studies were excluded for data duplication, methodological flaws and/or non-eligibility [25–27]. Of note, we did not find any trial investigating the use of the Chinese surfactant, while all other surfactants have been subjected to at least one trial. Characteristics of each included trial are summarized in the Additional file 1. We found 16 trials [28–43] but only 14 were finally included in the meta-analysis [28–41]. Five comment letters were also reviewed [44–48]. All studies compared a porcine surfactant with beractant, bovactant or BLES. No studies compared porcine surfactants with calfactant, Korean bovine surfactant or surfactant-TA.

Fifteen out of sixteen papers were in English, one was in Spanish [43] and was evaluated without translation, since two authors (AT,DDL) speak Spanish. Two studies [42, 43] were excluded from the meta-analysis because they investigated the use of non-internationally available porcine surfactants.

One of these surfactants is produced and only marketed in Cuba; the other one is a new, low-cost preparation only marketed in Brazil. The Brazilian surfactant has been compared versus controls treated with a mix of poractant-α and beractant [42]. The 14 studies included in the meta-analysis investigated poractant-α versus the above-mentioned bovine surfactants: five studies were conducted in North-America, five in Europe and four in Asia. A total of 1491 neonates were enrolled. Seven out of 14 studies were multicentre, although populations were relatively small (between 15 and 99 patients/arm). The use of antenatal steroids varied widely across the studies (from 25.9 to 100%), while all studies, apart from two, [37, 38] enrolled babies with mean gestational age ≤ 30 weeks. Two studies investigated the effect of poractant-α versus two distinct bovine surfactants in a three-arms design: [28, 37] data from the arms treated with bovine surfactants have been aggregated for the meta-analysis, since earlier meta-analysis did not detect significant differences between bovine surfactants [9]. All studies, apart from two, [28, 39] investigated poractant-α at the 200 mg/kg dose. One study [39] trialled beractant versus high dose (200 mg/kg) or low dose (100 mg/kg) poractant-α: data from 200 mg/kg arm have been used in the first analysis and data from both arms have been pooled together in the second analysis, irrespective of the dosage used.

Risk of bias evaluation is reported in the Additional file 1. The studies performed mostly well in completeness of outcome analysis and reporting, but generally suffered from performance bias resulting from lack of blinding for interventions and outcome assessments (apart from one [39]). The methods of randomization and allocation concealment were unclear for the majority of studies. There seemed to be no significant Funnel plot asymmetry as reported in Additional file 1 (*p* = 0.862, Peters’ test).

**Fig. 2:**
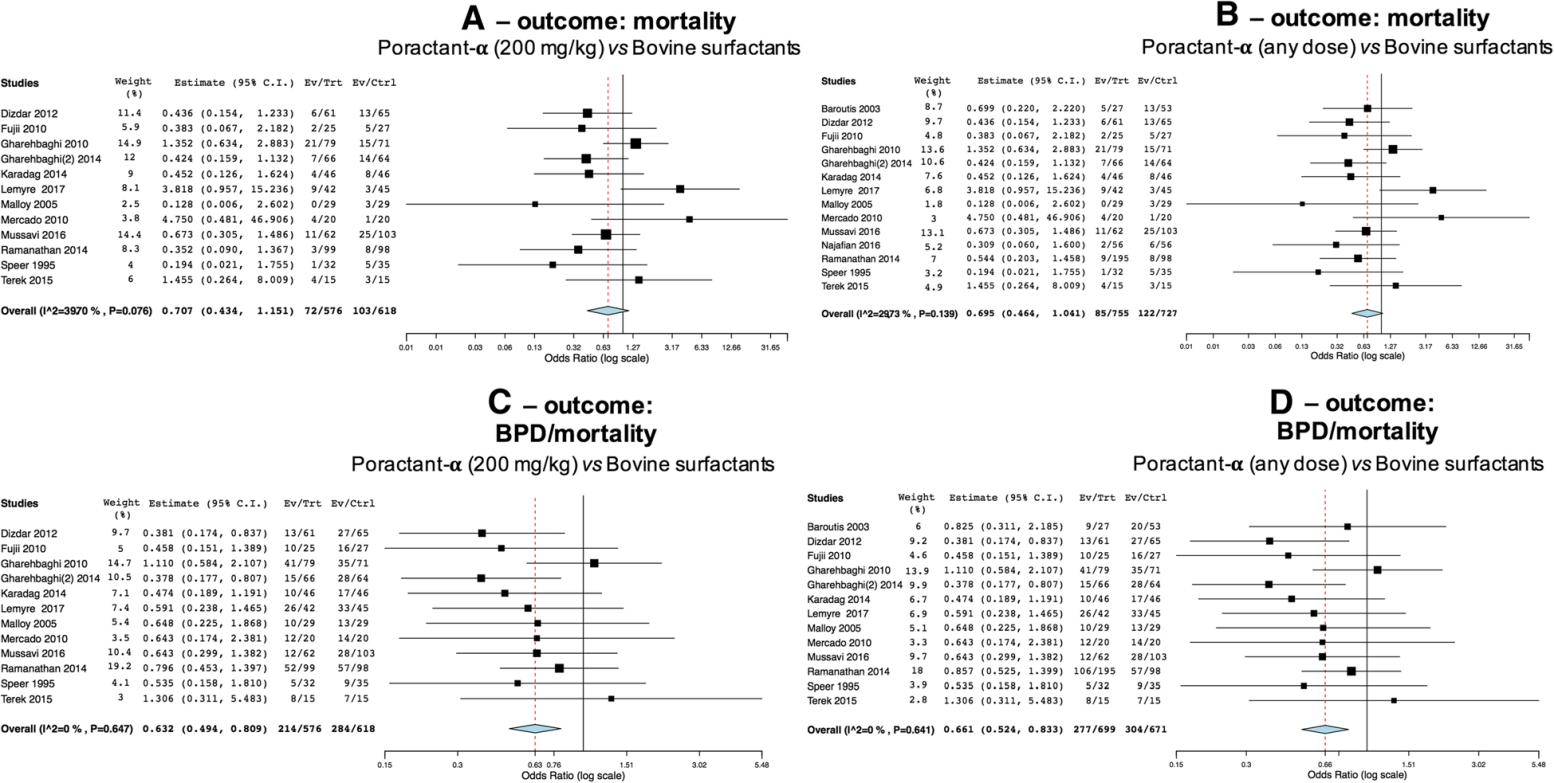
Comparisons poractant-α vs bovine surfactants for mortality (**c**-**b**) and for the composite outcome BPD/mortality (**c**-**d**). Panels **a**-**c** illustrate meta-analyses of 200 mg/kg poractant-α vs bovine surfactants (1193 patients); panels **b** (1482 patients) and **d** (1370 patients) illustrate meta-analyses of any dose of poractant-α vs bovine surfactants. Poractant-α and bovine surfactants are considered as treatment (Trt) and control (Ctrl) arm, respectively; events per arm and odds ratio (95%CI) are reported. All analyses have been performed with random effect model

The first and second analyses compared 200 mg/kg of poractant-α and any doses of poractant-α versus 100 mg/kg bovine surfactants, respectively: their results are very similar as the addition of trials irrespective of poractant-α dose only added 2–3 trials. Twelve trials reported mortality data studying 200 mg/kg poractant-α and 14 studying any dose of poractant-α. There appeared to be no significant differences between poractant-α and bovine surfactants in terms of mortality in both analyses (Fig. 2a: *p* = 0.164; Fig. 2b: *p* = 0.077). There is relevant heterogeneity within these studies: populations are probably not sufficiently large to reach statistical significance for this outcome. Twelve trials reported data on the composite BPD/mortality endpoint, studying poractant-α administered at a dose of 200 mg/kg and a further study evaluated this outcome studying poractant-α at a dose of 100 mg/kg. The incidence of this outcome is significantly lower in neonates treated with 200 mg/kg poractant-α (Fig. 2c: *p* < 0.001), and pooling together neonates treated with both doses (Fig. 2d: *p* < 0.001).

**Fig. 3:**
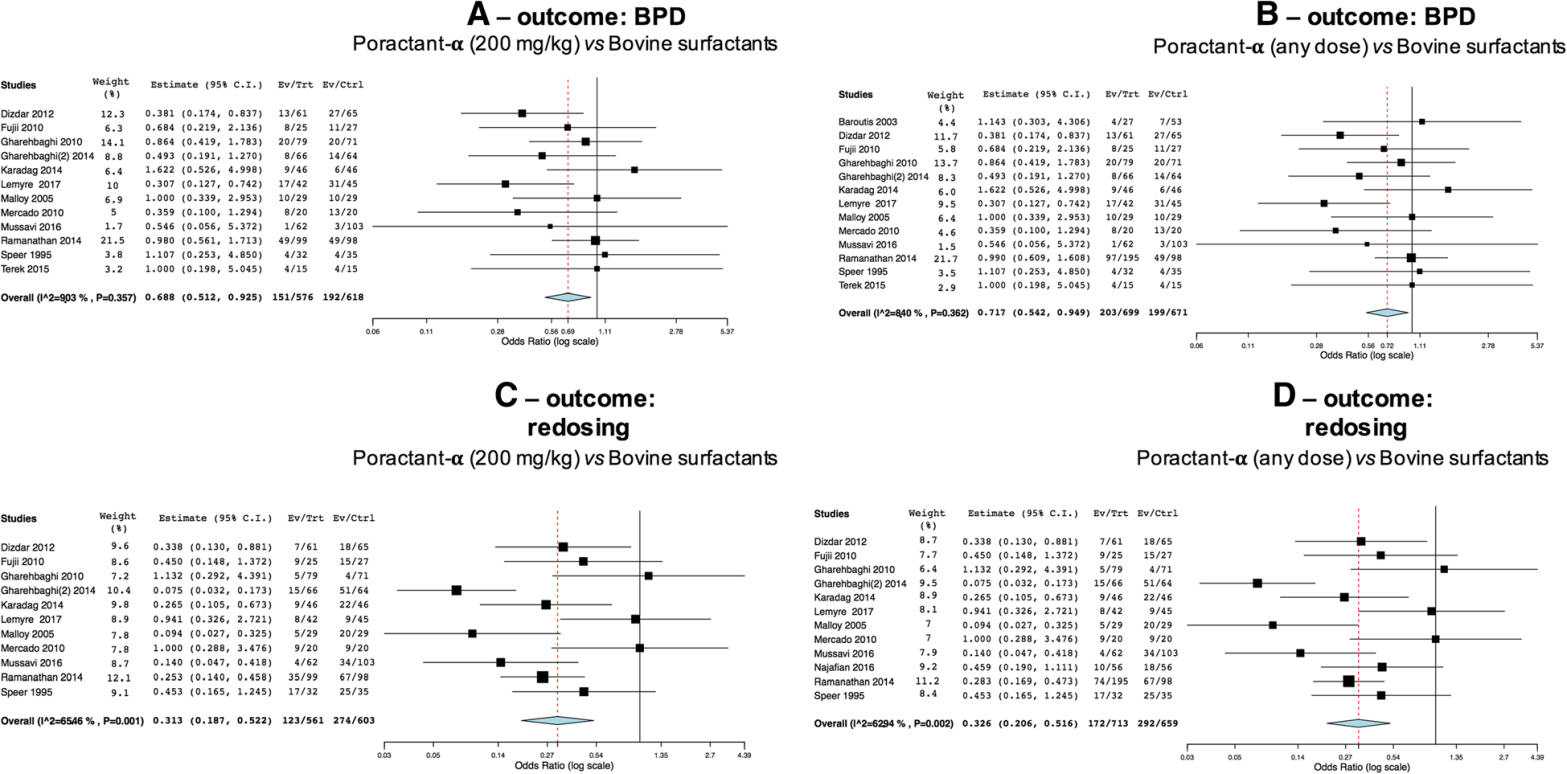
Comparisons poractant-α vs bovine surfactants for BPD (**a**-**b**) and surfactant redosing (**c**-**d**). Panels **a** (1193 patients) and **c** (1164 patients) illustrate meta-analyses of 200 mg/kg poractant-α vs bovine surfactants; panels **b** (1370 patients) and **d** (1372 patients) illustrate meta-analyses of any dose of poractant-α vs bovine surfactants. Poractant-α and bovine surfactants are considered as treatment (Trt) and control (Ctrl) arm, respectively; events per arm and odds ratio (95%CI) are reported. All analyses have been performed with random effect model

Poractant-α appeared associated with significantly lower incidence of BPD both when considering studies administering it at 200 mg/kg (Fig. 3a: *p* = 0.013) and when analyses were repeated pooling together patients receiving different doses (Fig. 3b: *p* = 0.019).

Data about surfactant redosing were available in 11 and 12 trials, for the 200 mg/kg and any dose, respectively. Poractant-α was associated with significantly lower incidence of surfactant retreatment in both analyses (Fig. 3c: *p* < 0.0001; Fig. 3d: *p* < 0.0001). Significant heterogeneity is evident for this comparison, probably due to the different criteria triggering surfactant retreatment used across the trials.

**Fig. 4:**
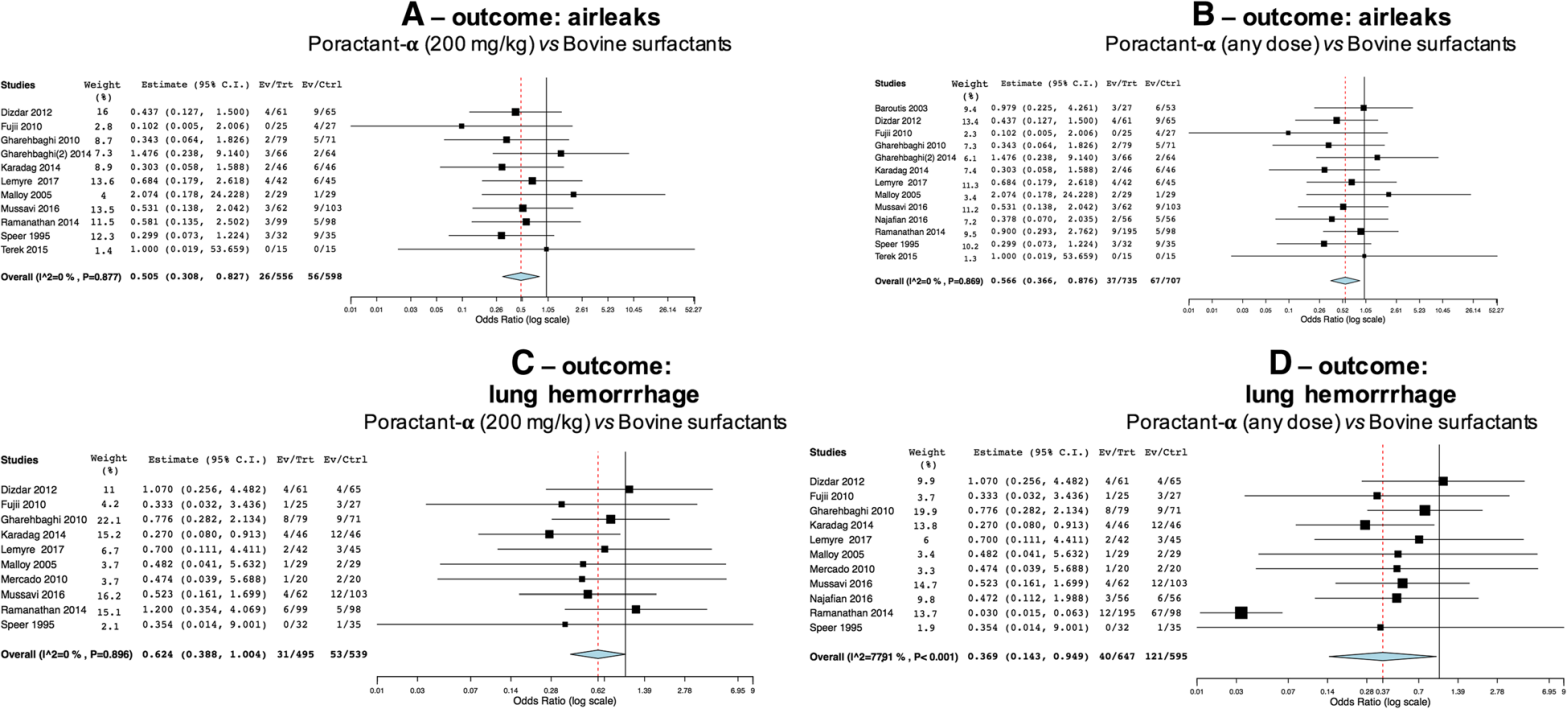
Comparisons poractant-α vs bovine surfactants for airleaks (**a**-**b**) and lung haemorrhage (**c**-**d**). Panels **a** (1154 patients) and **c** (1034 patients) illustrate meta-analyses of 200 mg/kg poractant-α vs bovine surfactants; panels **b** (1442 patients) and **d** (1242 patients) illustrate meta-analyses of any dose of poractant-α vs bovine surfactants. Poractant-α and bovine surfactants are considered as treatment (Trt) and control (Ctrl) arm, respectively; events per arm and odds ratio (95%CI) are reported. All analyses have been performed with random effect model

Eleven trials reported airleaks data, evaluating poractant-α at a dose of 200 mg/kg and 13 studying any dose of poractant-α, respectively. Poractant-α appeared associated with a significantly lower incidence of airleaks both at 200 mg/kg dosing (Fig. 4a: *p* = 0.006) and when pooling together the patients across arms with different dosing regimens (Fig. 4b: *p* = 0.01).

Data about pulmonary haemorrhage were available in ten and eleven trials for the analysis with 200 mg/kg poractant-α and any dose, respectively. There is a nearly significant reduction of pulmonary haemorrhage with the use of 200 mg/kg poractant-α (Fig. 4c: *p* = 0.051), while this reduction becomes significant when analysis was done pooling together patients receiving different doses of poractant-α (Fig. 4d: *p* = 0.038). Significant heterogeneity was detected only for the analysis of any dose of poractant-α versus bovine surfactants, due to the inclusion of one more study [38] in the analysis.

**Fig. 5:**
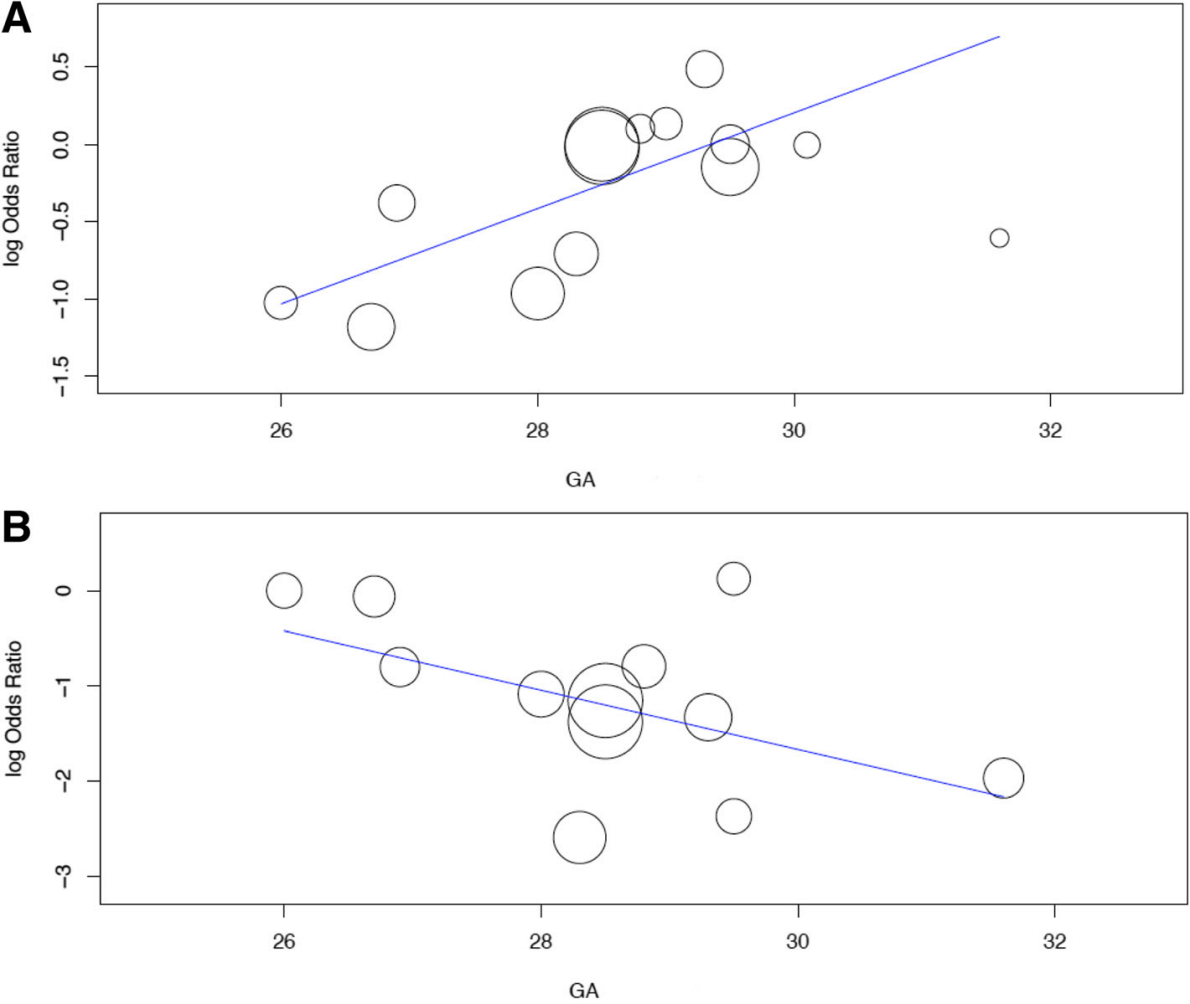
Meta-regressions plots. Gestational age (GA) – response relationships for the incidence of bronchopulmonary dysplasia (Panel **a**; the lower the gestational age, the lower the odds) and the need for surfactant retreatment (Panel **b**; the higher the gestational age, the lower the odds) are shown. Gestational age is expressed in weeks

Gestational age is significantly associated with effect size of poractant-α on BPD (coefficient: 0.308 (95% CI: 0.063, 0.554); *p* = 0.014) and on surfactant retreatment (coefficient: -0.311 (95% CI: -0.595, − 0.028); *p* = 0.031): the age – response relationships are shown in Fig. 5. Prenatal steroids and the dose of poractant-α are not significantly associated with effect size for any outcome comparison (*p* always > 0.05), as shown in the Additional file 1.

## Discussion

### Summary of evidence

Three porcine surfactants (butantan, poractant-α and surfacen) currently exist, but only poractant-α is internationally marketed and has been compared to bovine surfactants in several trials. Our meta-analysis compared for the first time the effects of poractant-α with all bovine surfactants, based on an aggregate sample larger than the ones used in previous meta-analyses [9, 49]. This has been possible because the meta-analysed studies compared poractant-α with the three more commonly available bovine surfactants (beractant, bovactant, BLES), which have been already deemed to be clinically equivalent [9]. This is not surprising given the biochemical and pharmacological similarities between bovine surfactants (Table 1). Moreover, we have been able to include four studies, [34, 37, 38, 41] that were still unpublished or under classification at the time of the latest Cochrane review [9].

Our meta-analysis is also much larger than that published by Singh et al., as they could only include five trials comparing poractant-α and only with beractant [49].

In summary, our findings show a trend for reduced mortality and significantly reduced incidence of BPD/mortality, BPD, airleaks, lung haemorrhage and need for retreatment in neonates treated with poractant-α at 200 mg/kg dose, compared to those treated with bovine surfactants. Our findings also showed that effect size for BPD is higher at the lowest gestational ages. Conversely, the effect size for surfactant redosing is higher at the highest gestational ages.

These results are only partially similar to those of earlier works but they are also stronger. In fact, previous meta-analyses: 1) included four trials (and about 400 neonates) less than us, which accounts for about 25% of the studied population; 2) analysed only one type of bovine surfactant [9, 49] or have been performed with a network design, due to the lack of comparisons between each surfactant; [50] 3) could not aggregate trials as we did, since equivalence between bovine surfactants had been not demonstrated yet; [9] 4) did not analyse the effect of possible confounders, such as antenatal steroids, gestational age or poractant-α dose; 5) did not compare the biochemical and pharmacological features out of each surfactant and did not consider the clinical results in light of the biological background. Ours was intended to be not only a statistical work but also a multidisciplinary project coupling clinical outcomes with their biological plausibility.

### Biological and physiopathological plausibility

Overall, the evidence is not sufficiently robust to determine if porcine or bovine surfactants are better in terms of mortality. There seems to be a trend in favour of poractant-α, but definite conclusions will require larger studies. In fact, when we increased the population by adding the few studies on 100 mg/kg poractant-α, the odds ratio was at the border of significance, despite the use of a lower dose. However, these were only three studies, based on a few babies and much larger studies are needed for such a complex outcome. In fact, for preterm neonates, who often remain hospitalized for several weeks or months, in-hospital mortality is an extremely complex outcome and may be influenced by other additional factors. This may explain the observed heterogeneity.

In fact, surfactants reduce surface tension, improving compliance and gas exchange. Thus, surfactants can reduce early mortality due to respiratory failure. However, it is unclear how surfactants could influence several non-pulmonary life-threatening complications (i.e.: intracranial haemorrhage, necrotizing enterocolitis, late-onset sepsis) in this highly vulnerable population [45, 51].

Our findings suggest that poractant-α (at 200 mg/kg) may be associated with lower incidence of BPD/mortality and BPD. This may be understandable as a more efficacious surfactant should reduce the duration of ventilation and its deleterious effects on the developing lung. We did not evaluate the effect of surfactants on the oxygenation, ventilatory parameters, ventilator-free days or similar measures, as these were inconsistently defined in the trials, and, when reported, summarized in different ways, making the data unsuitable for quantitative aggregation. Furthermore, ventilatory strategies significantly vary across different neonatal units [52].

Neonates treated with poractant-α (at 200 mg/kg) appeared to have better short-term respiratory outcomes compared to those treated with bovine surfactants, and, in particular, lower incidence of airleaks, lung haemorrhage and need for retreatment. These improved outcomes may be explained with a higher poractant-α effectiveness or may be due to the higher deliverable dose. It can be hypothesized that poractant-α may resist longer to hydrolysis and injury caused by secretory phospholipase A2 and other inflammatory agents, which are often present especially in extremely preterm neonates [53, 54]. As the mean time needed for preterm neonates to produce enough endogenous surfactant is about 4 days, [55] an exogenous surfactant able to remain effective for a longer time may not require re-treatment. Moreover, exogenous surfactant stimulates endogenous production [56] and an efficient surfactant should be also capable of reducing the need for aggressive ventilation, avoiding its pro-inflammatory effect [57].

The hypothesized higher efficiency of poractant-α may be related to two factors: 1) the phospholipid profile, as some minor phospholipids may protect surfactant from its catabolism; [58] 2) the hydrophobic protein content, especially for surfactant protein-B, which is needed to stabilize surfactant film and allow its spreading at the air/liquid interface and also to protect phospholipids from phospholipase- induced hydrolysis [59, 60]. While more basic research is needed to identify the best lipid profile, the role of surfactant proteins is well known, as older synthetic protein-free surfactants are clinically inferior to animal-derived surfactants, which at least carry some amounts of proteins [8].

The higher dose of poractant-α is also likely to explain these results. In fact, administering 200 instead of 100 mg/kg would provide lungs with more biophysically active molecules and enough surfactant may remain active in the alveoli, even in the presence of surfactant-injuring and inflammatory agents [54]. Pharmacokinetic and clinical data show that the 200 mg/kg poractant-α regimen should be preferred to the 100 mg/kg one, [6] since the higher dose provide a longer half life, less retreatment and a better clinical response in terms of oxygenation [61, 62]. It is important to note that higher doses are not easy to administer with bovine surfactants given their lower concentration and higher viscosity, [63] requiring higher volumes of administration: larger doses may cause lung oedema, potentially increasing the need for more aggressive ventilation and this may trigger a vicious cycle. The analysis for surfactant retreatment showed significant heterogeneity and this is possibly due to the different redosing criteria used in the different trials. This reflects the variability in practice existing across neonatal units, [64] hence the decision to analyse the data in a pragmatic way.

Effect sizes for BPD and surfactant retreatment are significantly associated with gestational age: poractant-α reduces more significantly the risk of BPD at the lowest gestational ages, while it is more effective in reducing need for redosing at highest gestational ages. Prenatal steroids use varied widely across the studies, however our analysis demonstrated that antenatal steroids are not associated with effect size for any outcome comparison. Thus, the superiority of one surfactant over the others does not seem to be related to steroid prophylaxis. Our main results were unchanged analysing any dose of poractant-α (i.e.: when we aggregated trials of low-dose poractant-α and when we performed meta-regression models adjusting for poractant-α dose). However, these analyses have been performed considering data from only 2–3 trials and few babies treated with low poractant-α dose [28, 38, 39]. Therefore, there is not enough evidence to determine whether the administration of poractant-α at a dose of 100 mg/kg provides advantages over bovine surfactants.

Our results are methodologically stronger than those provided by previous meta-analyses for the reasons described above. In particular, bovine surfactants have been previously demonstrated to be clinically equivalent, and this equipoise is understandable in the light of their composition (Table 1). Our findings are also novel as they allowed to advance knowledge in the field and answer the question “*what is the best surfactant at its licensed dose*?”. Relevant knowledge advancements provided by the present work are summarised in Table 2**.**

**Table 2:** Knowledge advancement on the topic

Outcome	Cochrane Meta-analysis 2015 [9]^a^	Present work
Mortality	Favours poractant-α (for some types of mortality)^b^	*Trend favouring poractant-α*
BPD	No difference	*Favours poractant-α*
Airleaks	No difference	*Favours poractant-α*
Lung haemorrhage	No difference	*Favours poractant-α*
BPD/mortality	Favours poractant-α	*Favours poractant-α*
Re-treatment	Unknown or favours poractant-α^c^	*Favours poractant-α*
Effect of confounder	Cochrane Meta-analysis 2015	Present work
Prenatal steroids	Unknown	Not significant
Gestational age	Unknown	*Influences effect size on BPD and need for retreatment*
Poractant-α dose	Unknown	Unknown

Conclusions from the present work and the earlier meta-analysis [9] are compared. New insights are in italic texts

^a^including both the comparisons lavage bovine and minced bovine *vs* minced porcine surfactant - ^b^ only for *in-hospital* mortality. The overall neonatal mortality did not present any difference - ^c^ This outcome was analysed only for the comparison bovine minced vs porcine minced surfactants

### Limitations

Although some of our results are statistically, biologically and clinically significant, they should be interpreted cautiously because of the following limitations. We have chosen outcomes easily defined and reported. The shared outcome definitions allow to aggregate data, but small differences are present across studies, such as the criteria for BPD definition or surfactant re-treatment. Thus, we decided to perform a pragmatic meta-analysis, as differences reflect the reality of neonatal care across hospitals using different BPD definitions and surfactant re-treatment criteria [65]. Moreover, studied populations were relatively small and the quality of the studies varied: the potential for bias was detected in almost every trial and this can impact on the results of meta-analysis.

We focused on short term respiratory outcomes as these are the easiest to define and are targeted in the majority of studies, although they may not necessarily be associated with long term respiratory outcomes [66]. However, these outcomes may represent life-threatening situations or be associated with significant burden of care.

As the majority of studies investigated poractant-α at 200 mg/kg, the evidence is not sufficiently robust to draw any conclusion about the low dose poractant-α. However, given the known clinical and pharmacokinetic advantages of 200 mg/kg dose of poractant-α, [61, 62] it might seem unethical to design a trial only to verify if the 200 mg/kg dose or the biochemical composition is responsible for the superiority of poractant-α. From the other hand a trial with doses higher than 100 mg/kg of bovine surfactant has never been conducted and would be almost technically impossible. In fact, as bovine surfactants are less concentrated, higher doses can cause lung edema requiring more aggressive ventilation. Hemodynamic troubles potentially leading to intracranial hemorrhage and other complications have already been described with usual doses of bovine surfactants [33]. For these reasons, also the more recent animal studies only investigated bovine surfactants at their licensed dose [67].

We did not perform an individual patient meta-analysis but rather meta-regressions and therefore some results might be subjected to the limitations of this technique, including some confounders that may have not been captured [22].

We did not perform analyses about any extra-pulmonary outcome, as this is the subject of another presently ongoing project. Moreover, it is a matter of debate how surfactant may influence these outcomes, especially in an era of antenatal steroids use and less invasive ventilation policies [45, 51].

We used a random-effects model which creates an average of different treatment effects, not an estimate of a single true treatment effect, and this may influence the interpretation of the results. This choice was however correct due to the relevant heterogeneity detected.

Finally, we based our work on the “clinical equivalence” of bovine surfactants according to the available evidence and to the biological/pharmacological differences between surfactants [9]. Combining different drugs into a single group may be seen as a limitation, although it is supported by the similar pharmacological and biochemical features of bovine surfactants. These features were not totally new. However, data about surfactant pharmacological and biochemical properties were dispersed in several papers or industrial databases and not always fully or easily available to the readers. Our work in this area consisted in collecting these data, make them comparable and easily available as much as possible and finally linked to the clinical data obtained by the meta-analysis. This last work had never been done and it is important to fully understand the available clinical evidence.

## Conclusions

Poractant-α (at 200 mg/kg dose) is associated with better short term respiratory outcomes when compared to bovine surfactants *at their licensed dose* in preterm neonates with RDS. The effect of poractant-α in terms of BPD reduction is greater at lowest gestational age, while the effect on the need for retreatment is greater at highest gestational ages.

## Additional file

Additional file 1: **Table S1.** Characteristics of studies included in the systematic review and in the meta-analysis. **Figure S1.** Results of bias assessment. **Table S2.** Additional meta-regression results. (DOCX 2973 kb)

## Abbreviations

BPD: bronchopulmonary dysplasia
ESPR: European Society for Paediatric Research
OR: odds ratio
PAS: Paediatric Academic Societies
RDS: Respiratory distress syndrome
