# Supplementary material for: Porcine vs bovine surfactant therapy for preterm neonates with RDS: systematic review with biological plausibility and pragmatic meta-analysis of respiratory outcomes
Source: Respir Res. 2019 Feb 6;20:28. doi: 10.1186/s12931-019-0979-0 (PMC6366095; doi:10.1186/s12931-019-0979-0)
Supplement: Supplementary file 1 — Table S1. Characteristics of studies included in the systematic review and in the meta-analysis. Figure S1. Results of bias assessment. Table S2. Additional meta-regression results. (DOCX 2973 kb) [file 12931_2019_979_MOESM1_ESM.docx]

**Additional file 1**

**Porcine *vs* bovine surfactant therapy for preterm neonates with RDS: systematic review with biological plausibility and meta-analysis of respiratory outcomes**

**Ascanio Tridente** (MD,PhD), **Lucia De Martino** (MD) and **Daniele De Luca** (MD,PhD)

**Table S1.** Characteristics of studies included in the systematic review and in the meta-analysis. **Pag.2-4**

**Figure S1.** Results of bias assessment. **Pag.5**

**Table S2.** Additional meta-regression results. **Pag.6**

**References Pag.7**

**Additional file 1: Table S1. Characteristics of studies included in the systematic review and in the meta-analysis.** Two studies were excluded from the pragmatic meta-analysis because they investigated the use of two non-internationally marketed porcine surfactants.

**§** All studies eligible for the review and meta-analysis enrolled preterm neonates with clinical and/or radiologic evidence of RDS: the additional inclusion criteria selected patients based on their clinical severity, gestational age and/or birth weight.

**#** refers to the mean gestational age of the whole trial population (as weighted mean gestational age of the different arms).

**^** RDS score is the so called “Downes' score”, which considers gestational age, respiratory rate, breath sounds on auscultation, grunting, retractions and FiO_2_ with possible scores ranging from 0 to 12. (9)

**†** Ramanathan’s study (14) trialled beractant versus high dose (200 mg/kg) or low dose (100 mg/kg) poractant-α. Thus, data from these arms were handled separately in the analyses cording to the dose, or pooled together for the analysis irrespective of the dosage used.

**n.a.:** indicates data not available (not described in the paper and not provided by the authors asked for)

Studies with an asterisk (*****) have a multicentre design.

**Abbreviations**: a/A: arterial/alveolar ratio; BPD: Broncho-pulmonary dysplasia; BW: birth weight; FiO_2_: inspired oxygen fraction; GA: gestational age; OI: oxygenation index (Paw x FiO_2_/PaO_2_); PaO_2_: arterial partial pressure of oxygen; Paw: mean airway pressure; RDS: respiratory distress syndrome; SatO_2_: peripheral arterial oxygen saturation.

| **Studies included in the meta-analysis** | | | | | | | |
| --- | --- | --- | --- | --- | --- | --- | --- |
| **Author/year (ref.)** | **Country** | **Comparison (n° of patients)** | **Dose**  **(mg/kg)** | **Additional inclusion criteria §** | **Antenatal steroids (%/arm)** | **GA#**  **(weeks)** | **Outcomes** |
| Baroutis/2003 (1)  commented in (2) | Greece | Bovactant (27) *vs* Poractant-α (27) *vs* Beractant (26) | 100 *vs* 100 *vs* 100 | BW≤2000g - FiO_2_>0.3  (within 24h of life) | 33% - 26% - 31% | 29 ± 0.9 | Mortality, BPD, airleaks |
| Dizdar/2012 (3) | Turkey | Poractant-α (61)  *vs* Beractant (65) | 200 *vs* 100 | FiO_2_≥0.30 (within 6h of life) | 61% - 51% | 28 ± n.a. | Mortality, BPD, surfactant re-dosing, airleaks, lung haemorrhage |
| Fuji/2010* (4)  commented in (5) | USA | Poractant-α (25)  *vs* Beractant (27) | 200 *vs* 100 | GA<30wks - Need for mechanical ventilation with Paw≥6 cmH_2_O and FiO_2_>30% (within 8h of life) | 100% - 96% | 26.9 ± 1.6 | Mortality, BPD, surfactant re-dosing, airleaks, lung haemorrhage |
| Gharehbaghi/2010* (6) | Iran | Poractant-α (79)  *vs* Beractant (71) | 200 *vs* 100 | None | 45% - 42% | 29.5 ± 2.8 | Mortality, BPD, surfactant re-dosing, airleaks, lung haemorrhage |
| Gharehbaghi/2014* (7) | Iran | Poractant-α (66)  *vs* Bovactant (64) | 200 *vs* 100 | GA≤32wks - FiO_2_>30%  (within 6h of life) | 62% - 53% | 28.3 ± 2.1 | Mortality, BPD, surfactant re-dosing, airleaks |
| Karadag/2014 (8) | Turkey | Poractant-α (46)  *vs* Beractant (46) | 200 *vs* 100 | GA≤32wks – Need for mechanical ventilation with Paw>7 cmH_2_O and FiO_2_>0.4 to have PaO_2_=70-80 mmHg (within 2h of life) | 76% - 83% | 29.3 ± 1.8 | Mortality, BPD, surfactant re-dosing, airleaks, lung haemorrhage |
| Lemyre/2017* (9) | Canada | Poractant-α (42)  *vs* BLES (45) | 200 *vs* 135 | GA<32wks (within 48h of life) - RDS score^ >8 or >1 of the following: 1)  FiO_2_≥0.4; 2) pH<7.20 and PaCO_2_> 60mmHg (persistent & associated with clinical distress) or 3) recurrent apnea | 67% - 73% | 26.7 ± 1.9 | Mortality, BPD, surfactant re-dosing, airleaks, lung haemorrhage |
| Malloy/2005 (10) | USA | Poractant-α (29)  *vs* Beractant (29) | 200 *vs* 100 | Need for surfactant as per clinical judgment and unit policy | 69% - 79% | 29.5 ± 3.2 | Mortality, BPD, surfactant re-dosing, airleaks, lung haemorrhage |
| Mercado/2010 (11) | USA | Poractant-α (20)  *vs* Beractant (20) | 200 *vs* 100 | GA<30wks | 95% - 95% | 26 ± 1.5 | Mortality, BPD, surfactant re-dosing, lung haemorrhage |
| Mussavi/2016 (12) | Iran | Bovactant (49) *vs* Poractant-α (62) *vs* Beractant (54) | 100 *vs* 200 *vs* 100 | Within 6h of life | 92% - 91% - 87% | 31.6 ± 3.7 | Mortality, BPD, surfactant re-dosing, airleaks, lung haemorrhage, |
| Najafian/2016* (13) | Iran | Poractant-α (56)  *vs* Beractant (56) | 100 *vs* 100 | BW>750g - SatO_2_ between 85% and 96% - GA<35wks (within 6h of life) | n.a. | 32.6 ± 3.3 | Mortality, surfactant re-dosing, airleaks, lung haemorrhage, |
| Ramanathan/2004* † (14)  commented in (15) | USA | Hi-dose Poractant-α (99)  *vs* Low-dose Poractant-α (96) *vs* Beractant (98) | 200 *vs* 100 *vs* 100 | BW between 750 and 1750g – Need for mechanical ventilation with FiO_2_≥0.30 or a/A≤33 (within 6h of life) | 76% - 82% - 85% | 28.5 ± 2 | Mortality, BPD, surfactant re-dosing, airleaks, lung haemorrhage |
| Speer/1995* (16)  commented in (17) | Germany | Poractant-α (33)  *vs* Beractant (40) | 200 *vs* 100 | BW between 700 and 1500g - Need for mechanical ventilation with FiO_2_≥0.40 (within 24h of life) | 42% - 37% | 28.8 ± 2.2 | Mortality, BPD, surfactant re-dosing, airleaks, lung haemorrhage |
| Terek/2015 (18)  commented in (19) | Turkey | Poractant-α (15)  *vs* Beractant (18) | 200 *vs* 100 | GA between 26 and 36wks - FiO_2_/SpO_2_<0.22 or FiO_2_>0.4 or OI>7 (within 2h of life) | 72% - 80% | 30.1 ± 3.3 | Mortality, BPD, airleaks |
| **Studies not included in the meta-analysis** | | | | | | | |
| **Author/year** | **Country** | **Comparison (patients)** | **Dose**  **(mg/kg)** | **Additional inclusion criteria §** | **Antenatal steroids (%/arm)** | **GA (weeks)** | **Reason for exclusion from meta-analysis** |
| Rebello/2014 (20) | Brazil | Butantan (154) vs Poractant-α or Beractant (173) | 100 *vs* 100 | BW≥500g; postnatal age -  PaO_2_/FiO_2_≤175 (within 24h of life) | 51% - 54% | 28 ± 2 | Butantan not commercially available worldwide.  Control arm treated with a mix of Poractant-α or Beractant: impossible to separate data |
| Sanchez-Mendiola/2005 (21) | Mexico | Surfacen (21) vs Beractant (23) | 100 *vs* 100 | FiO_2_>0.5 and Paw>8 cmH_2_O to have PaO_2_>60 mmHg (within 8h of life) | n.a. | 31 ± 3.3 | Surfacen not commercially available worldwide. |

**Additional file 1: Figure S1. Results of bias assessment: risk of bias for each trial (A) and publication bias (B).** Panel A shows the risk of bias evaluation performed with the Cochrane Risk of Bias assessment tool (26). Each item was assessed as at “low”(+, green squares) or “high risk” (-, red squares) of bias, or unclear (?, yellow squares), when the investigators were unable to determine, on the basis of the available data).

Panel B shows Funnel plot. This is a scatter plot of the studies in a meta-analysis (blue dots) in a space defined by effect size (on the x-axis; scale displayed on top of the plot) and standard error (on the y-axis). It also presents the combined effect size (CES; green dot) with its confidence interval (black). The plot also shows a vertical line (also in red) that runs through the (adjusted) combined effect size and the corresponding lower and upper limits of the confidence interval (red diagonal lines). The diagonal hatched line represents the line generated by the Egger regression. (23) The adjusted combined effect size and accompanying confidence and prediction intervals in this plot represents the results of a trim-and-fill procedure as proposed by Duval and Tweedie. (24,25)

**
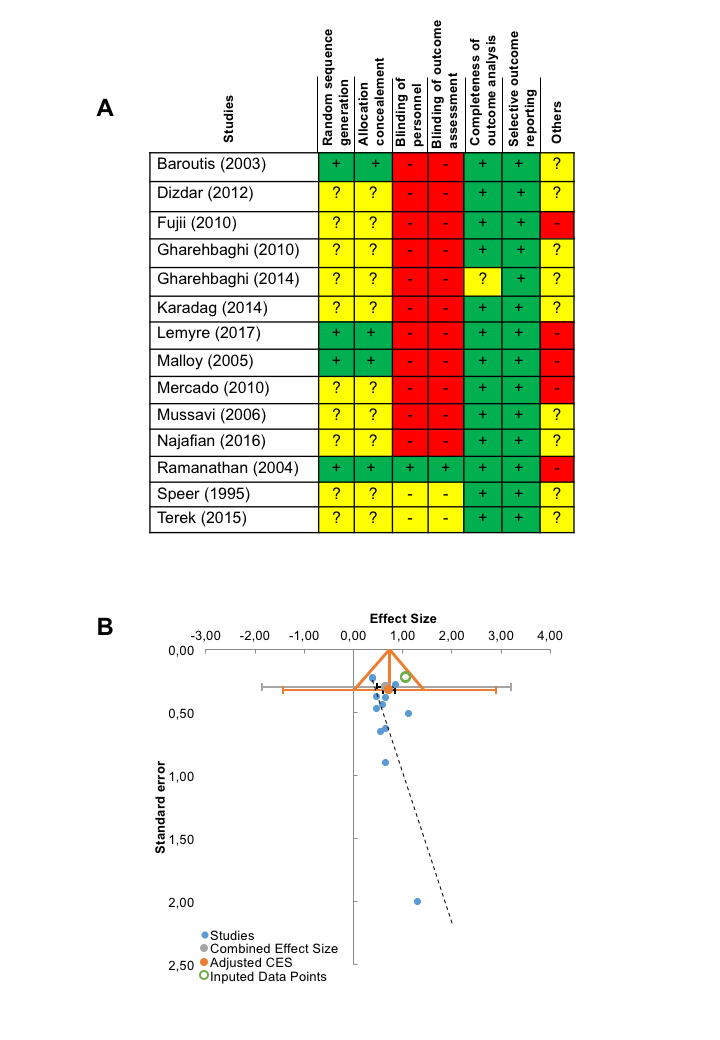
**

**Table S2. Results of meta-regression when adjusting for antenatal steroids or poractant-α dose.** Antenatal steroids (as % of neonates treated in each trial) and poractant-α dose (100 or 200 mg/kg) have been studied as confounders; coefficients (and 95%CI) are shown.

|  | **Coefficient**  **(95% CI)** | **p** | **Model goodness-of-fit** |
| --- | --- | --- | --- |
| **Mortality** | | | 0.965 |
| Antenatal steroids | 0.001  (-0.014, 0.017) | 0.861 |  |
| Poractant-α dose | 0.02  (-0.943, 0.986) | 0.965 |  |
| **BPD/Mortality** |  |  | 0.429 |
| Antenatal steroids | 0.002  (-0.008, 0.012) | 0.728 |  |
| Poractant-α dose | 0.349  (-0.2, 0.899) | 0.213 |  |
| **BPD** |  |  | 0.325 |
| Antenatal steroids | 0.005  (-0.007, 0.016) | 0.442 |  |
| Poractant-α dose | 0.351  (-0.24, 0.94) | 0.246 |  |
| **Surfactant redosing** |  |  | 0.983 |
| Antenatal steroids | -0.002  (-0.023, 0.019) | 0.855 |  |
| Poractant-α dose | 0.047  (-1.36, 1.45) | 0.948 |  |
| **Airleaks** |  |  | 0.311 |
| Antenatal steroids | 0.004  (-0.015, 0.023) | 0.694 |  |
| Poractant-α dose | 0.808  (-0.25, 1.87) | 0.136 |  |
| **Lung Haemorrhage** |  |  | 0.384 |
| Antenatal steroids | -0.01  (-0.03, 0.01) | 0.354 |  |
| Poractant-α dose | 0.821  (-0.52, 2.16) |  |  |

**References for additional material**

1.[Baroutis G](https://www.ncbi.nlm.nih.gov/pubmed/?term=Baroutis%20G%5BAuthor%5D&cauthor=true&cauthor_uid=12709796), [Kaleyias J](https://www.ncbi.nlm.nih.gov/pubmed/?term=Kaleyias%20J%5BAuthor%5D&cauthor=true&cauthor_uid=12709796), [Liarou T](https://www.ncbi.nlm.nih.gov/pubmed/?term=Liarou%20T%5BAuthor%5D&cauthor=true&cauthor_uid=12709796), [Papathoma E](https://www.ncbi.nlm.nih.gov/pubmed/?term=Papathoma%20E%5BAuthor%5D&cauthor=true&cauthor_uid=12709796), [Hatzistamatiou Z](https://www.ncbi.nlm.nih.gov/pubmed/?term=Hatzistamatiou%20Z%5BAuthor%5D&cauthor=true&cauthor_uid=12709796), [Costalos C](https://www.ncbi.nlm.nih.gov/pubmed/?term=Costalos%20C%5BAuthor%5D&cauthor=true&cauthor_uid=12709796).Comparison of three treatment regimens of natural surfactant preparations in neonatal respiratory distress syndrome.

Eur J Pediatr 2003;162:476-80.

2.[Shalwitz RA](https://www.ncbi.nlm.nih.gov/pubmed/?term=Shalwitz%20RA%5BAuthor%5D&cauthor=true&cauthor_uid=14628142). Comparison of treatment regimens of natural surfactant preparations in neonatal respiratory distress syndrome. Eur J Pediatr 2004;163:126;

3. [Dizdar EA](https://www.ncbi.nlm.nih.gov/pubmed/?term=Dizdar%20EA%5BAuthor%5D&cauthor=true&cauthor_uid=22105435), [Sari FN](https://www.ncbi.nlm.nih.gov/pubmed/?term=Sari%20FN%5BAuthor%5D&cauthor=true&cauthor_uid=22105435), [Aydemir C](https://www.ncbi.nlm.nih.gov/pubmed/?term=Aydemir%20C%5BAuthor%5D&cauthor=true&cauthor_uid=22105435), et al. A randomized, controlled trial of poractant alfa versus beractant in the treatment of preterm infants with respiratory distress syndrome. Am J Perinatol 2012;29:95-100.

4. [Fujii AM](https://www.ncbi.nlm.nih.gov/pubmed/?term=Fujii%20AM%5BAuthor%5D&cauthor=true&cauthor_uid=20336076), [Patel SM](https://www.ncbi.nlm.nih.gov/pubmed/?term=Patel%20SM%5BAuthor%5D&cauthor=true&cauthor_uid=20336076), [Allen R](https://www.ncbi.nlm.nih.gov/pubmed/?term=Allen%20R%5BAuthor%5D&cauthor=true&cauthor_uid=20336076), [Doros G](https://www.ncbi.nlm.nih.gov/pubmed/?term=Doros%20G%5BAuthor%5D&cauthor=true&cauthor_uid=20336076), [Guo CY](https://www.ncbi.nlm.nih.gov/pubmed/?term=Guo%20CY%5BAuthor%5D&cauthor=true&cauthor_uid=20336076), [Testa S](https://www.ncbi.nlm.nih.gov/pubmed/?term=Testa%20S%5BAuthor%5D&cauthor=true&cauthor_uid=20336076). Poractant alfa and beractant treatment of very premature infants with respiratory distress syndrome. J Perinatol 2010;30:665-70.

5. Lutchman D. Have we been down this road before? J Perinatol 2010;30:698-9;

6. [Gharehbaghi MM](https://www.ncbi.nlm.nih.gov/pubmed/?term=Gharehbaghi%20MM%5BAuthor%5D&cauthor=true&cauthor_uid=20589459), [Sakha SH](https://www.ncbi.nlm.nih.gov/pubmed/?term=Sakha%20SH%5BAuthor%5D&cauthor=true&cauthor_uid=20589459), [Ghojazadeh M](https://www.ncbi.nlm.nih.gov/pubmed/?term=Ghojazadeh%20M%5BAuthor%5D&cauthor=true&cauthor_uid=20589459), [Firoozi F](https://www.ncbi.nlm.nih.gov/pubmed/?term=Firoozi%20F%5BAuthor%5D&cauthor=true&cauthor_uid=20589459). Complications among premature neonates treated with beractant and poractant alfa. Indian J Pediatr 2010;77:751-4.

7. [Gharehbaghi MM](https://www.ncbi.nlm.nih.gov/pubmed/?term=Gharehbaghi%20MM%5BAuthor%5D&cauthor=true&cauthor_uid=20589459), Yasrebi S. Comparing the Efficacy of two Natural Surfactants, Curosurf and Alveofact, in Treatment of Respiratory Distress Syndrome in Preterm Infants.

Int J Women Health Reprod Sci 2014;2:245-248.

8. [Karadag N](https://www.ncbi.nlm.nih.gov/pubmed/?term=Karadag%20N%5BAuthor%5D&cauthor=true&cauthor_uid=24566756), [Dilli D](https://www.ncbi.nlm.nih.gov/pubmed/?term=Dilli%20D%5BAuthor%5D&cauthor=true&cauthor_uid=24566756), [Zenciroglu A](https://www.ncbi.nlm.nih.gov/pubmed/?term=Zenciroglu%20A%5BAuthor%5D&cauthor=true&cauthor_uid=24566756), [Aydin B](https://www.ncbi.nlm.nih.gov/pubmed/?term=Aydin%20B%5BAuthor%5D&cauthor=true&cauthor_uid=24566756), [Beken S](https://www.ncbi.nlm.nih.gov/pubmed/?term=Beken%20S%5BAuthor%5D&cauthor=true&cauthor_uid=24566756), [Okumus N](https://www.ncbi.nlm.nih.gov/pubmed/?term=Okumus%20N%5BAuthor%5D&cauthor=true&cauthor_uid=24566756). Perfusion index variability in preterm infants treated with two different natural surfactants for respiratory distress syndrome. Am J Perinatol 2014;31:1015-22.

9. [Lemyre B](https://www.ncbi.nlm.nih.gov/pubmed/?term=Lemyre%20B%5BAuthor%5D&cauthor=true&cauthor_uid=28472058), [Fusch C](https://www.ncbi.nlm.nih.gov/pubmed/?term=Fusch%20C%5BAuthor%5D&cauthor=true&cauthor_uid=28472058), [Schmölzer GM](https://www.ncbi.nlm.nih.gov/pubmed/?term=Schm%C3%B6lzer%20GM%5BAuthor%5D&cauthor=true&cauthor_uid=28472058), et al. Poractant alfa versus bovine lipid extract surfactant for infants 24+0 to 31+6 weeks gestational age: A randomized controlled trial. PLoS One 2017;12:e0175922.

10. [Malloy CA](https://www.ncbi.nlm.nih.gov/pubmed/?term=Malloy%20CA%5BAuthor%5D&cauthor=true&cauthor_uid=16188788), [Nicoski P](https://www.ncbi.nlm.nih.gov/pubmed/?term=Nicoski%20P%5BAuthor%5D&cauthor=true&cauthor_uid=16188788), [Muraskas JK](https://www.ncbi.nlm.nih.gov/pubmed/?term=Muraskas%20JK%5BAuthor%5D&cauthor=true&cauthor_uid=16188788). A randomized trial comparing beractant and poractant treatment in neonatal respiratory distress syndrome. Acta Paediatr 2005;94:779-84.

11. [Mercado VV](https://www.ncbi.nlm.nih.gov/pubmed/?term=Mercado%20VV%5BAuthor%5D&cauthor=true&cauthor_uid=20586001), [Cristea I](https://www.ncbi.nlm.nih.gov/pubmed/?term=Cristea%20I%5BAuthor%5D&cauthor=true&cauthor_uid=20586001), [Ali N](https://www.ncbi.nlm.nih.gov/pubmed/?term=Ali%20N%5BAuthor%5D&cauthor=true&cauthor_uid=20586001), et al. Does surfactant type cause a differential proinflammatory response in preterm infants with respiratory distress syndrome? Adv Ther 2010;27:476-82.

12. [Mussavi M](https://www.ncbi.nlm.nih.gov/pubmed/?term=Mussavi%20M%5BAuthor%5D&cauthor=true&cauthor_uid=28203337), [Mirnia K](https://www.ncbi.nlm.nih.gov/pubmed/?term=Mirnia%20K%5BAuthor%5D&cauthor=true&cauthor_uid=28203337), [Asadollahi K](https://www.ncbi.nlm.nih.gov/pubmed/?term=Asadollahi%20K%5BAuthor%5D&cauthor=true&cauthor_uid=28203337). Comparison of the Efficacy of Three Natural Surfactants (Curosurf, Survanta, and Alveofact) in the Treatment of Respiratory Distress Syndrome Among Neonates: A Randomized Controlled Trial. Iran J Pediatr 2016;26:e5743.

13. Najafian B, Karimi-Sari H, Hossein Khosravi M, Nikjoo N, Amin S, Shohrati M. Comparison of efficacy and safety of two available natural surfactants in Iran, Curosurf and Survanta in treatment of neonatal respiratory distress syndrome: A randomized clinical trial. Contemp Clin Trials Commun 2016;3:55-59.

14. [Ramanathan R](https://www.ncbi.nlm.nih.gov/pubmed/?term=Ramanathan%20R%5BAuthor%5D&cauthor=true&cauthor_uid=15085492), [Rasmussen MR](https://www.ncbi.nlm.nih.gov/pubmed/?term=Rasmussen%20MR%5BAuthor%5D&cauthor=true&cauthor_uid=15085492), [Gerstmann DR](https://www.ncbi.nlm.nih.gov/pubmed/?term=Gerstmann%20DR%5BAuthor%5D&cauthor=true&cauthor_uid=15085492), [Finer N](https://www.ncbi.nlm.nih.gov/pubmed/?term=Finer%20N%5BAuthor%5D&cauthor=true&cauthor_uid=15085492), [Sekar K](https://www.ncbi.nlm.nih.gov/pubmed/?term=Sekar%20K%5BAuthor%5D&cauthor=true&cauthor_uid=15085492); [North American Study Group](https://www.ncbi.nlm.nih.gov/pubmed/?term=North%20American%20Study%20Group%5BCorporate%20Author%5D). A randomized, multicenter masked comparison trial of poractant alfa (Curosurf) versus beractant (Survanta) in the treatment of respiratory distress syndrome in preterm infants. Am J Perinatol 2004;21:109-19.

15. Bancalari E. On "A randomized, multicenter masked comparison trial of poractant alfa (Curosurf) versus beractant (Survanta) in the treatment of respiratory distress syndrome in preterm infants" (Am J Perinatol 2004;21:109-120). Am J Perinatol 2004;21:307-9.

16. Speer CP, Gefeller O, Groneck P, et al. [Randomised clinical trial of two treatment regimens of natural surfactant preparations in neonatal respiratory distress syndrome.](https://www.ncbi.nlm.nih.gov/pubmed/7743295) Arch Dis Child Fetal Neonatal Ed 1995;72:F8-13.

17. Logan S. Need to avoid bias in controlled trials. Arch Dis Child Fetal Neonatal Ed 1995;73:F121.

18. Terek D, Gonulal D, Koroglu OA, Yalaz M, Akisu M, Kultursay N. [Effects of Two Different Exogenous Surfactant Preparations on Serial Peripheral Perfusion Index and Tissue Carbon Monoxide Measurements in Preterm Infants with Severe Respiratory Distress Syndrome.](https://www.ncbi.nlm.nih.gov/pubmed/25603725) Pediatr Neonatol 2015;56:248-55.

19. Jeng MJ. Effects of Two Different Exogenous Surfactant Preparations on Serial Peripheral Perfusion Index and Tissue Carbon Monoxide Measurements in Preterm Infants with Severe Respiratory Distress Syndrome.

Pediatr Neonatol 2015;56:211-212.

20. Rebello CM, Precioso AR, Mascaretti RS, Grupo Colaborativo do Estudo Brasileiro Multicêntrico de Surfactante. A multicenter, randomized, double-blind trial of a new porcine surfactant in premature infants with respiratory distress syndrome. Einstein (Sao Paulo) 2014;12:397–404.

21. Sánchez-Mendiola M, Martínez-Nater OC, Herrera-Maldonado N, Ortega-Arroyo J. Estudio controlado del tratamiento de la enfermedad de membrana hialina del recién nacido pretérmino con surfactante pulmonar exógeno (porcino vs. bovino). Gac Méd Méx 2005;4:267-271. [article in Spanish]

22. Higgins JP, Altman DG, Gøtzsche PC, et al; Cochrane Bias Methods Group; Cochrane Statistical Methods Group. [The Cochrane Collaboration's tool for assessing risk of bias in randomised trials.](https://www.ncbi.nlm.nih.gov/pubmed/22008217) BMJ 2011;343:d5928.

23. [Sterne JA](https://www.ncbi.nlm.nih.gov/pubmed/?term=Sterne%20JA%5BAuthor%5D&cauthor=true&cauthor_uid=21784880), [Sutton AJ](https://www.ncbi.nlm.nih.gov/pubmed/?term=Sutton%20AJ%5BAuthor%5D&cauthor=true&cauthor_uid=21784880), [Ioannidis JP](https://www.ncbi.nlm.nih.gov/pubmed/?term=Ioannidis%20JP%5BAuthor%5D&cauthor=true&cauthor_uid=21784880), et al. Recommendations for examining and interpreting funnel plot asymmetry in meta-analyses of randomised controlled trials. BMJ 2011;343:d4002.

24. Duval S, Tweedie R. A nonparametric "trim and fill" method of accounting for publication bias in meta-analysis. J Am Stat Assoc 2000;95: 89-98.

25. Duval S. Tweedie R. Trim and fill: A simple funnel-plot-based method of testing and adjusting for publication bias in meta-analysis. Biometrics 2000;56:455-463.
